# Hydronephrosis: An Unusual Complication of a Diverticular Abscess

**DOI:** 10.7759/cureus.31016

**Published:** 2022-11-02

**Authors:** Aalam Sohal, Hunza Chaudhry, Lin Li

**Affiliations:** 1 Internal Medicine, Liver Institute Northwest, Fresno, USA; 2 Internal Medicine, University of California, San Francisco Fresno, Fresno, USA

**Keywords:** complications, renal, hydronephrosis, diverticular abscess, acute diverticulitis

## Abstract

Acute diverticulitis is a common medical condition with known complications such as abscess, perforation, obstruction, and fistula formation, among others. Ureteral complications such as hydronephrosis have rarely been reported in the literature. We report the case of a 40-year-old man with diverticulitis found to have a perisigmoid abscess complicated by left-sided hydronephrosis. It is essential to consider this entity in patients with right lower quadrant pain to ensure prompt diagnosis and treatment.

## Introduction

Acute diverticulitis is a prevalent gastrointestinal disorder marked by inflammation due to microperforation of the diverticulum in the colon [1]. It is a condition frequently encountered in primary care and emergency department settings. It is a highly prevalent disease with 2.7 million outpatient visits and 200,000 inpatient admissions at a cost of 1.2 billion [2]. It is commonly seen in elderly patients; however, recent studies have shown a growing incidence in patients younger than 50 [3,4]. Acute diverticulitis can be uncomplicated or complicated depending on abscess formation, fistula, or frank perforation [2]. Although ureteral complications from gastrointestinal diseases such as inflammatory bowel disease and colon cancer have been extensively documented in the literature, hydronephrosis as a complication of diverticulitis has rarely been reported [3,5,6]. Here, we present the case of a 40-year-old man with diverticulitis complicated by an abscess found to have left-sided hydronephrosis.

## Case presentation

**Figure 1:**
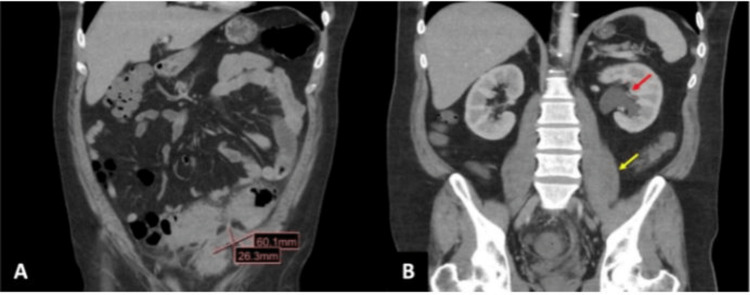
(A) Sigmoid diverticulitis with perisigmoid abscess measuring 6 cm × 2.6 cm. (B) Left-sided hydroureter (yellow arrow) and hydronephrosis (red arrow) noted on the computed tomography scan of the abdomen with contrast.

A 40-year-old man with a medical history of laparoscopic cholecystectomy presented to the emergency department with left lower quadrant cramping abdominal pain for two weeks. He endorsed constipation for the last three months but denied other symptoms. He also reported using laxatives with no improvement in his symptoms. On presentation, he was afebrile and hemodynamically stable. His physical examination was significant for a palpable phlegmon in the left lower quadrant and suprapubic area with mild tenderness to palpation. Laboratory values revealed hemoglobin of 11.4 g/dL, platelet count of 499 k/µL, and unremarkable basic metabolic panel. Computed tomography (CT) scan of the abdomen with contrast revealed severe sigmoid diverticulitis with adjacent fat stranding and perisigmoid abscess measuring 6 × 2.6 cm (Figure 1A). It further revealed moderate left hydronephrosis with narrowing of the ureter, concerning for external ureteral compression by the diverticular abscess (Figure 1B).

Interventional radiology was consulted, and they were unable to drain the abscess as no amenable window was present for drainage. After multidisciplinary discussion with interventional radiology, urology, and surgery, the recommendation was made to undergo surgical intervention after the resolution of acute diverticulitis. The patient was treated with intravenous antibiotics with symptomatic relief and discharged with a plan for outpatient surgery.

Unfortunately, the patient was lost to follow-up. He presented three months later with perforated diverticulitis, persistent hydronephrosis, colovesicular and colocutaneous fistula requiring exploratory laparotomy, Hartmann’s procedure, ileocolectomy, and bladder repair. His surgical pathology was negative for malignancy and revealed severe diverticulitis. The patient was discharged home in stable condition.

## Discussion

Ureteral complications have been documented in patients with advanced colon cancer and inflammatory bowel disease [3,4]. This is mainly seen due to the close anatomical location of the left ureter to the descending and sigmoid colon. Common urologic complications include the development of enterovesical fistulas, urinary calculi, and ureteral obstruction. The incidence of hydroureteronephrosis secondary to acute diverticulitis is unclear. A study by Siminovitch et al. in 1980 on 400 patients admitted with diverticulitis revealed that 5% of the patients had hydroureteronephrosis based on urograms [7]. On a review of the literature, there are scant reports documenting hydronephrosis as a complication of sigmoid diverticulitis [8-13].

None of these cases reported the development of an abscess that led to hydronephrosis. Flowers et al. documented a case of bilateral hydronephrosis secondary to sigmoid diverticulitis and a complicated diverticular abscess [13]. Another case reported segment colitis associated with diverticulosis as a cause of persistent hydronephrosis [14]. This case brings to our attention the lesser-known fact that a diverticular abscess can also lead to the development of hydronephrosis.

Acute uncomplicated diverticulitis can be managed, inpatient or outpatient. Patients who are immunocompromised, cannot tolerate oral intake, or have peritonitis should be managed in the inpatient setting as they require intravenous antibiotics and fluids. Conservative measures with bowel rest and increased fluid intake can be pursued for patients on an outpatient basis. Our patient had complicated diverticulitis with the development of a diverticular abscess measuring 6 cm × 2.6 cm. Percutaneous drainage is preferred in patients with abscesses greater than or equal to 4 cm [15]. Studies show that the overall success rate of nonoperative management for a diverticular abscess is 80%, regardless of approaches, while the remaining 20% need surgical intervention [16]. Surgery was not an option for our patient due to the location of the abscess; instead, our patient was treated conservatively with antibiotics.

Unfortunately, the patient’s clinical course was complicated by perforated bowel requiring surgical resection and placement of end-colostomy. This also raises the question of whether hydronephrosis or ureteral involvement is a sign of impending perforation and whether patients with such presentations should undergo surgical resection. Literature is scarce regarding the conservative management of these patients as the majority of previously reported cases of diverticulitis had surgical resection [7-12]. Further retrospective studies are required to study the incidence and impact of hydronephrosis on the outcomes of patients with diverticulitis.

## Conclusions

Our case highlights a rare complication of the diverticular abscess. The close proximity of the sigmoid colon to the ureter may result in the development of hydronephrosis. Physicians should be aware of this association, and further studies are needed to study the impact of this complication on the clinical course of patients.
